# Autoimmunity as a Driving Force of Cognitive Evolution

**DOI:** 10.3389/fnins.2017.00582

**Published:** 2017-10-26

**Authors:** Serge Nataf

**Affiliations:** CarMeN Laboratory, Bank of Tissues and Cells, Institut National de la Santé et de la Recherche Médicale 1060, INRA 1397, INSA Lyon, Lyon University Hospital (Hospices Civils de Lyon), Université Claude Bernard Lyon-1, Lyon, France

**Keywords:** autoimmunity, cognitive evolution, T-cell repertoire, autism, schizophrenia, paraneoplastic syndromes, Alzheimer's disease, Parkinson's disease

## Abstract

In the last decades, increasingly robust experimental approaches have formally demonstrated that autoimmunity is a physiological process involved in a large range of functions including cognition. On this basis, the recently enunciated “brain superautoantigens” theory proposes that autoimmunity has been a driving force of cognitive evolution. It is notably suggested that the immune and nervous systems have somehow co-evolved and exerted a mutual selection pressure benefiting to both systems. In this two-way process, the evolutionary-determined emergence of neurons expressing specific immunogenic antigens (brain superautoantigens) has exerted a selection pressure on immune genes shaping the T-cell repertoire. Such a selection pressure on immune genes has translated into the emergence of a finely tuned autoimmune T-cell repertoire that promotes cognition. In another hand, the evolutionary-determined emergence of brain-autoreactive T-cells has exerted a selection pressure on neural genes coding for brain superautoantigens. Such a selection pressure has translated into the emergence of a neural repertoire (defined here as the whole of neurons, synapses and non-neuronal cells involved in cognitive functions) expressing brain superautoantigens. Overall, the brain superautoantigens theory suggests that cognitive evolution might have been primarily driven by internal cues rather than external environmental conditions. Importantly, while providing a unique molecular connection between neural and T-cell repertoires under physiological conditions, brain superautoantigens may also constitute an Achilles heel responsible for the particular susceptibility of *Homo sapiens* to “neuroimmune co-pathologies” i.e., disorders affecting both neural and T-cell repertoires. These may notably include paraneoplastic syndromes, multiple sclerosis as well as autism, schizophrenia and neurodegenerative diseases. In the context of this theoretical frame, a specific emphasis is given here to the potential evolutionary role exerted by two families of genes, namely the MHC class II genes, involved in antigen presentation to T-cells, and the Foxp genes, which play crucial roles in language (Foxp2) and the regulation of autoimmunity (Foxp3).

## Introduction

Evolution of the Homo genus is essentially characterized by a process of encephalization that has accompanied the acquisition of unique cognitive skills. As compared to non-human primates, one of the main features of the human brain appears to reside on a functionally powerful synaptic plasticity allowing the acquisition and maintenance of highly complex cognitive skills. In humans, the development and maturation of fully functional synaptic circuits requires a particularly long period of time, elapsing from fetal life to late adolescence (Miller et al., 2012; van de Leemput et al., 2014; Whitaker et al., 2016). The construction of such a neuronal circuitry is grounded on evolutionary-determined programs and shaped by environmental cues whose impact is also elapsing from fetal life to late adolescence. Interestingly, plasticity and developmental dependence to environmental cues are similarly two major hallmarks of the immune system, at least in vertebrates. This is worth noting since the nervous and immune systems are the two best-performing biological apparatus that vertebrates dispose of for the perception of and adaption to the external environment. The nervous and immune systems share multiple functional commonalities that were first highlighted by Irun Cohen's seminal papers describing the “cognitive immune system” (Cohen, 1992a,b). Many authors further emphasized such similarities and I personally attempted to nourish the cognitive immunology paradigm by bringing about the notion of immune objects as immune counterparts of visual objects (Nataf, 2014, 2016). However, besides functional analogies, there is possibly a particular evolutionary link that bridges the nervous and immune systems. Such a connection was recently suggested to rely, among other mechanisms, on the evolutionary-determined emergence of autoimmune responses directed against a specific set of brain autoantigens (Nataf, 2017b). This theoretical framework referred to as the “brain superautoantigens” theory states that cognitive evolution may have been driven, at least in part, by autoimmunity against brain antigens (Nataf, 2017b). Furthermore, such a process of autoimmunity-directed cognitive evolution is proposed to have been responsible for the particular susceptibility of *Homo sapiens* to a wide array of human “neuro-immune co-pathologies” (Nataf, 2017a,b). Indeed, there is compelling evidence that the immune and nervous systems are concurrently affected in disorders that appear to be, if not specific to humans, at least much more frequent in *Homo sapiens* than in non-human primates. These notably include organ-specific autoimmune diseases (Wagner et al., 2001; Vierboom et al., 2005; Aliesky et al., 2013; 't Hart, 2016) neurodegenerative conditions (Capitanio and Emborg, 2008) and psychiatric disorders such as autism and schizophrenia (Ogawa and Vallender, 2014). A first question that may arise from such a view is: what evolutionary advantage would confer a selection pressure exerted jointly on the immune and nervous systems? Before answering this question, it might be helpful to recall that the concept of symbiosis, beyond its classical meaning in the context of inter-species interactions, currently embodies all the inter-cellular interactions governing homeostasis, equilibrium and harmony at the scale of a tissue (Gray, 2017; Tauber, 2017). By extension, “symbiosis” between tissues as well as “symbiosis” between systems are hallmarks of a physiological regulation of the internal milieu at the scale of a whole organism. In this regard, one has to point that symbiosis between the immune and nervous systems is likely to be of particular importance. This assumption is supported by the previously mentioned observation that both systems are endowed with a unique ability to perform an intelligent sensing of and adaptation to the external environment. In line with this general frame, 3 major statements listed below summarize the brain superautoantigens theory and the associated “co-development co-evolution” model:

- in a large range of species, the central nervous system co-develops with the immune system
- the immune and nervous systems as well as their symbiotic relationships have co-evolved across species and have reached their highest levels of complexity in *Homo sapiens*
- the mutual selection pressure exerted by the neural and T-cell repertoires has allowed a species-specific endogenously-driven adaptation to the external environment.

Of note, the notion of a neuro-immune symbiosis is in full agreement with the recently enunciated theory of an immune-mediated evolution of linguistic skills (Benítez-Burraco and Uriagereka, 2016). However, a major issue is to precisely identify the molecular pathways supporting such a symbiotic neuroimmune interaction. A triangular evolutionary-determined symbiosis between gut microbiota, brain and the immune system represents an attractive hypothesis (Benítez-Burraco and Uriagereka, 2016). Another explanation, not exclusive from the former, is that autoimmunity against “brain superautoantigens” fuels a direct molecular connection between the T-cell and neural repertoires, as defined and explained in the following sections. Below are several prerequisites that need to be exposed, notably to non-immunologists or non-neuroscientists, before going more deeply into this theoretical frame. A short lexicon of the basic neuroimmunology terms used in sections Immune Repertoires and Autoimmunity and The Notion of Neural Repertoire is presented in Table 1.

**Table 1:** Short lexicon of basic neuroimmunology terms.

Adaptive immunity	***Immunity** resulting from the **recognition of antigens***
Innate immunity	***Immunity** resulting from **non-specific activating signals***
T-cells	***T lymphocytes:** cells that recognize antigens*
B-cells	***B lymphocyte:** cells that secrete antibodies*
TH cells	***T helper cells:** subset of T lymphocytes that orchestrate adaptive immunity*
TH1 cells	***T helper 1 cells:** subset of pro-inflammatory TH cells secreting interferon-γ*
TH17 cells	***T helper 17 cells:** subset of pro-inflammatory TH cells secreting IL-17*
Antigens	***Peptides recognized** by T-cells and/or antibodies*
Autoantigens	***≪Self≫ peptides recognized** by T-cells and/or antibodies*
TCRs	***T-cell receptors:** T-cell membranous receptors recognizing antigens*
APCs	***Antigen-presenting cells:** immune cells that capture, process and present antigens*
MHC molecules	***Major histocompatibility complex molecules:** the molecular pockets of antigens*
HLA molecules	***Human leukocyte antigens:** human MHC molecules*
HLA-DR molecules	***HLA-DR:** a subtype of HLA class II molecules*
Neural cells	***Cells of the nervous system***
Neuronal cells	***Neurons***
Neurites	***Cytoplasmic expansions** that extend from the **cell body of neurons***
Axons	***Unique type of neurites** supporting **nerve conduction***
Myelin sheath	***Lipid-rich** sheath that **insulates axons***
Oligodendrocytes	***Neural cells** that synthesize **myelin sheaths***
Astrocytes	***Neural cells** supporting **the survival and functions of neurons***
Synapses	***Structures supporting** the interneuronal transmission of nerve impulses*
Synpatic plasticity	***Establishment, elimination, strengthening or weakening of synapses***

## Immune repertoires and autoimmunity

### Adaptive immunity as an immune counterpart of the learning brain

The term “adaptive immunity” designates the whole of immune cells and molecules that supports finely-tuned immune responses directed against specific antigens i.e., specific immune targets. In mammals, B-lymphocytes (the antibody-secreting cells) and T-lymphocytes represent the great majority of adaptive immune cells. Each T-lymphocyte bears a unique receptor called T-cell receptor (TCR) that is able to recognize a specific target antigen (more precisely an antigen-derived peptide; see below). The number of distinct T-lymphocytes bearing distinct TCRs is estimated to reach 2.5 × 10^7^ in humans (Arstila et al., 1999, 2000). The term “T-cell repertoire” designates thus the whole of distinct TCRs bore by T-cells at a given time in a given individual. To use a common military metaphor, this means that during the whole life span of an individual, the needs to combat enemies from the outside (infectious agents) or from within (cancer cells) are fulfilled by an army of highly efficient soldiers that can recognize and eventually eliminate as much as 2.5 × 10^7^ different antigens (targets) (Arstila et al., 1999, 2000). Besides the T-cell repertoire, the antibody repertoire encompasses the whole of distinct antigen-specific antibodies that are secreted by B-cells at a given time in a given individual. Thus, immune repertoires include the T-cell repertoire and the antibody repertoire. Contrasting with “adaptive immunity,” the so-called “innate immunity” relies on cells that do not belong to the T- or B-lymphocyte lineages. Innate immune cells are endowed with little ability to discriminate between targets. Accordingly, only a limited number of such targets, under the form of molecular patterns, may activate innate immune cells in a specific manner. Finally, an important feature of adaptive immunity is the capacity to generate long-lived memory cells that engage a fast-acting recall program when re-exposed to their target antigen. Thus, just as the learning brain, the immune system exhibits a particular adaptive ability allowing: (i) the generation of output signals that are exquisitely adapted to a large array of distinct input stimuli, (ii) the learning and memory of a multitude of input-specific programs from which output responses can be appropriately recalled and modulated over time. Also, just as the learning brain, the adaptive ability of the immune system is framed and biased in a species-specific manner. This point is notably illustrated by the existence of so-called public TCRs i.e., TCRs that can be demonstrated in any individual belonging to a given species (see below) (Madi et al., 2014; Covacu et al., 2016).

### The dichotomy between “self” and “non-self” antigens

**Figure 1:**
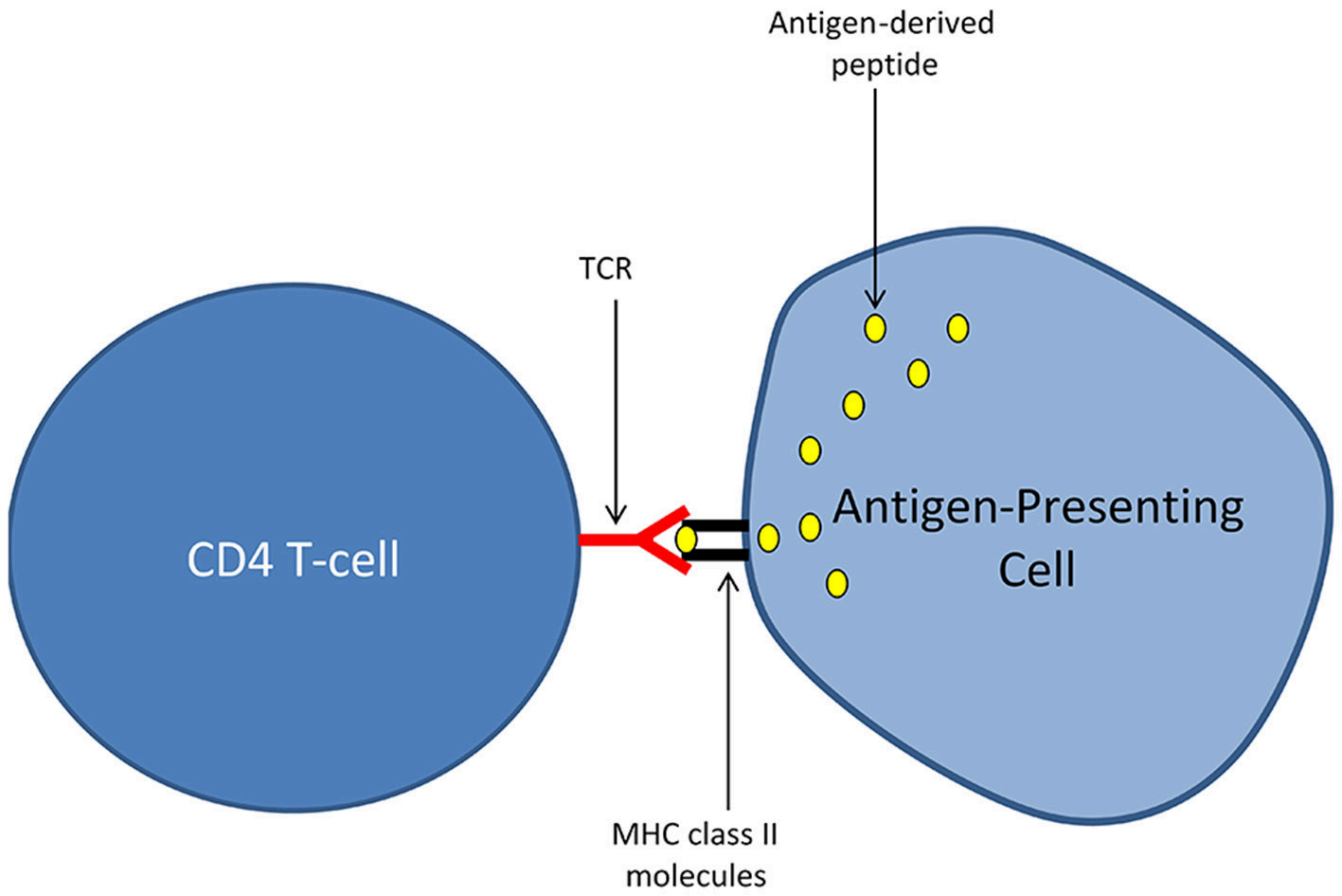
Presentation of antigen-derived peptides to T-cells. The activation of CD4 T-cells in an antigen-specific manner requires a process called antigen presentation and performed by specialized immune cells named antigen-presenting cells (APCs). APCs harbor unique abilities to capture extracellular antigens, to process (i.e., cleave) these antigens into short peptides and to expose at the outer surface of their cellular membrane the peptides generated by antigen processing. There, the exposed peptides are “recognized” by T-cells. Importantly, the recognition of antigen-derived peptides by T-cells also requires that such peptides physically associate with molecules of the major histocompatibility complex class II (MHC class II) that are expressed by APCs. The antigen-specific activation of a CD4 T-cell engages only if the TCR (T-cell receptor) expressed at its cell surface binds with a high affinity the molecular complex formed by: (i) a peptide derived from the targeted antigen and (ii) MHC class II molecules into which the antigen-derived peptide is loaded.

Again, for those who are not familiar with the immune system, I briefly expose bellow some of the basic mechanisms that are governing adaptive immune responses mediated by T-lymphocytes. This subsection is limited to a particular T-cell population, the “CD4 T-cells” (or TH cells for T helper cells), considered as the main orchestrators of adaptive immunity. Antigens, which can be defined as proteins harboring the potential to elicit an adaptive immune response, are not recognized by CD4 T-cells in their native form. T-cell activation in response to antigen recognition requires a process called antigen presentation that is performed by specialized immune cells named antigen-presenting cells (APCs). APCs exhibit unique abilities to capture extracellular antigens, to process (i.e., cleave) these antigens into short peptides and to expose at the outer surface of their cellular membrane the peptides generated by such an antigen processing. There, the exposed peptides are “recognized” by T-cells. Importantly, the recognition of antigen-derived peptides by T-cells also requires that such peptides physically associate with molecules of the major histocompatibility complex class II (MHC class II) that are expressed by APCs. In humans, MHC class II molecules are named Human Leucocyte Antigen (HLA) class II molecules and are encoded by a group of genes that are highly polymorphic. Any given antigen-derived peptide presented by an APC in association with the host's MHC molecules (i.e., HLA molecules in humans) is susceptible to induce an antigen-specific activation of T-cells. Such an antigen-specific T-cell activation occurs only in T-cells harboring an *ad hoc* T-cell receptor (TCR). Conversely, not all TCRs, and thus not all T-cells, recognize a given antigen-derived peptide. At the molecular level, the antigen-specific activation of a CD4 T-cell requires that the TCR on its cell surface binds with a high affinity the complex formed by: (i) a peptide derived from the targeted antigen and (ii) MHC class II molecules into which the antigen-derived peptide is loaded (Figure 1). MHC class II molecules are thus frequently depicted as the molecular pockets in which antigen-derived peptides locate. Deciphering the molecular mechanisms of antigen-specific T-cell activation has been a major advance in fundamental immunology (Marx, 1980). However, a crucial question quickly arose from this milestone discovery: how the immune system is coping with the risks of autoimmunity that are inherently linked to the ability of T-cells to recognize basically any antigen? The first answer to this question came from the notion of “non-self” antigens, a term that now designates the whole range of antigens that are not strictly deriving from the host's cells. Such “non-self” antigens notably comprise all microbial antigens. In this functional scheme, all the T-cells directed against “self” antigens are eliminated by a process of selection that essentially takes place in the thymus. As a consequence, only T-cells directed against “non-self” antigens persist in blood and tissues, allowing thus the immune system to efficiently tackle infections while avoiding pathological autoimmunity. Many aspects of this principle, primarily enunciated by Franck Macfarlane Burnet and coined as the “clonal selection theory” (Burnet, 1962; Raju, 1999) have been challenged in the last decades. In particular, the danger theory, put forward by Matzinger (1994), conceptualized experimental data showing that the immune system may tolerate “non-self” antigens as far as such antigens are not recognized in a context of danger or harmfulness. This is notably the case for antigens deriving from the commensal flora or from the fetus, which by nature fall both into the category of “non self” antigens. Another conceptual revolution came from the demonstration by Irun Cohen that autoimmunity i.e., immunity against “self” antigens is indeed a physiological process fulfilling a large range of protective functions (Cohen, 1988; Cohen and Young, 1991; Schwartz and Cohen, 2000). Thus, autoreactive T-cells may escape from the thymic selection process and exert physiological trophic functions as well immunosurveillance tasks against tumors (which express altered “self” antigens) (Schwartz and Cohen, 2000; Madi et al., 2015). Nevertheless, it was also suggested that physiological autoimmunity may pave the ground for pathological autoimmunity (Cohen, 2000). Furthermore, the very concept of antigen-specific T-cell activation was challenged whilst the notion of antigenic cross-reactivity emerged. Indeed, a single TCR may bind with a high affinity a large range of unique peptides loaded in the MHC class II molecules of the host. Most importantly, immune defense against microbial antigens was shown to essentially rely on a mechanism of cross-reactivity between “self” and “non-self” antigens (Wucherpfennig et al., 2007; Mandl et al., 2013; Fulton et al., 2015; Quinn et al., 2016). Along this line, a large share of species-specific public TCRs were shown to recognize “self” antigens (Madi et al., 2014, 2017).

### From physiological autoimmunity to cognition-promoting autoimmunity

While, in the late 80s, physiological autoimmunity was initially envisioned by Irun Cohen as a pathway allowing the immune system to sense tissue integrity (Cohen, 1988; Cohen and Young, 1991), a cognition-promoting role of natural autoimmunity was demonstrated later on by Michal Schwartz and Jonathan Kipnis starting from the mid-2000s (Schwartz and Kipnis, 2001, 2011; Ziv et al., 2006; Derecki et al., 2010; Kipnis et al., 2012; Schwartz et al., 2013; Kipnis, 2016). Notably, in elegant transfer experiments, T-cell deficient mice were shown to exhibit profound cognitive alterations that could be alleviated by replenishment with autoreactive CD4 T-cells directed against brain autoantigens (Ziv et al., 2006). T-cell clones directed against myelin basic protein (MBP), a major component of myelin sheaths (the insulating structure that enwraps nerve fibers and facilitates nerve conduction) were shown to support the proliferation of neural stem/progenitor cells via the synthesis of neurotrophic factors (essentially neurotrophins i.e., a family of molecules that support the survival of neurons and/or the proliferation/differentiation of neural stem/progenitors cells) (Hauben et al., 2000; Moalem et al., 2000; Yoles et al., 2001). Other studies demonstrated then that cognition-promoting T-cells are indeed memory T-cells that can be demonstrated in the following specific locations: (i) the meninges (envelopes surrounding the CNS) (Derecki et al., 2010; Kipnis et al., 2012; Radjavi et al., 2014) and the choroid plexuses (interfaces between the blood and the cerebrospinal fluid) (Baruch and Schwartz, 2013; Kunis et al., 2013) i.e., the two histological structures forming the borders of the central nervous system (CNS) and (ii) the cervical lymph nodes (Radjavi et al., 2014) i.e., the immune organs in which immune cells that migrate from or toward the brain are interacting (Calzascia et al., 2005; Raper et al., 2016). Accordingly, while cognition-promoting autoimmunity was initially thought to require direct contacts between T-cells and neurons or their progenitors, it is now demonstrated that such a T-cell mediated effect is essentially performed at distance from neurons in anatomical sites located outside the CNS (cervical lymph nodes and other lymphoid organs) (Radjavi et al., 2014; Niebling et al., 2017) or at the CNS borders (meninges, choroid plexuses) (Derecki et al., 2010; Kipnis et al., 2012; Baruch and Schwartz, 2013; Kunis et al., 2013; Radjavi et al., 2014). An important question arising from the concept of cognition-promoting autoimmunity is to determine by which pathway(s) the immune system may be exposed to brain autoantigens. Owing to the lack lymphatic vessels (vessels specialized in the transport of immune cells and antigens) in the CNS parenchyma, the circulation of cells and antigens from the CNS toward the periphery (where immune organs locate) has been long lastly considered as extremely limited. However, lymphatic vessels were indeed demonstrated in the meninges and were shown to support the trafficking of cells and antigens from the CNS to the cervical lymph nodes (Raper et al., 2016). Moreover, CNS-derived antigens contained in microvesicles called exosomes were also demonstrated in blood (Shi et al., 2014; García-Romero et al., 2017) exposing thus the immune system to brain autoantigens. Other potential pathways are described in details elsewhere (Nataf, 2017a). The capture of brain superautoantigens by APCs may be notably complemented by the constitutive expression of brain superautoantigens by APCs (i.e., the apparently inappropriate transcription of neural genes in immune cells).

Another important point relates with the phenotype of brain-autoreactive and neuroprotective T-cell clones. While T-cells secreting anti-inflammatory molecules would have been intuitively considered as a unique source of cognition-promoting autoreactive T-cells, several works demonstrated that cognition-promoting T-cells may also exhibit pro-inflammatory phenotypes. Indeed the cytokine profiles of cognition-promoting T-cells may be similar to those of pathogenic autoreactive T-cells involved in autoimmune disorders. Thus, cognition-promoting T-cells includes a substantial share of pro-inflammatory brain-autoreactive T-cells. Such a population notably comprises so-called TH1 (T Helper 1) and TH17 (T Helper 17) cells i.e., subsets of TH cells that essentially secrete interferon-γ and IL-17A (interleukin 17A) respectively (Moalem et al., 2000; Kunis et al., 2013; Niebling et al., 2017). Along this line, accumulating experimental data show that, irrespective of their cellular source, pro-inflammatory cytokines such as TNF-α (tumor necrosis alpha)(Beattie et al., 2002) and interferon-γ exert major physiological functions in the developing or mature brain (Zhu et al., 2011; Filiano et al., 2016). Overall, an appropriately-controlled level of inflammation appears indispensable to the physiological functioning of the CNS (Xanthos and Sandkühler, 2013).

### Functional repertoires superimpose to the TCR-determined T-cell repertoire

While the term T-cell repertoire is used to depict the array of antigen-specific TCRs bore by the whole T-cell population, one should bear in mind that, beyond antigen recognition, the heterogeneity of T-cells is determined by other crucial properties. T-cells sharing a common TCR may be heterogeneous in terms of life span, cytokine profile and/or homing properties (i.e., ability to migrate in specific anatomical sites). There are thus functional repertoires that superimpose to the TCR repertoire and are essential to the overall orchestration of T-cell-mediated immunity. Accordingly, cognition-promoting autoimmunity is likely to be afforded by a subset of brain-autoreactive T-cells that not only recognize brain antigens but express a specific functional signature with regard to cytokine/neurotrophin synthesis and homing properties. Any quantitative or qualitative distortion of such a unique brain-autoreactive T-cell population is likely to engender a risk of “neuroimmune co-pathology” (see section Neuroimmune Co-pathologies). Of note, we mentioned above that the TCR repertoire is determined by experience but also by species-specific and evolutionary-determined immune programs. Thus, evolutionary genetics plays an important role in the TCR repertoire of an individual. In a similar manner, evolutionary genetics is likely to be a major determinant of the functional T-cell repertoires bore by an individual. This is likely to be notably the case for the expression of neurotrophins by T-cells.

## The notion of neural repertoire

In a wide array of species, the nervous system can be viewed as a central processor that integrates complex inputs deriving from the external or internal environment. The integration of such inputs is required for an *ad hoc* neurally-mediated adaptation to both external and internal milieus. The neural output signals triggered by the perception of endogenous or exogenous inputs can be schematically divided into the following two main categories: (i) signals that generate actions, essentially motor actions, via communications with non-neural cells, (ii) signals that do not generate actions and stay confined within the nervous system. Importantly, what best characterize the nervous system is a particular mode of information transfer, called synaptic transmission, allowing extremely fast and long distance communications between neurons. Thus, neurons of whatever species share neuron-specific morphological and phenotypic features that mostly serve to support the propagation of electrical and/or chemical signals along specialized cytoplasmic expansions called neurites (i.e., dendrites and axons, the two categories of neurites). However, a yet not fully resolved issue resides in the identification of mechanisms which dictate the nervous system speciation. It now clearly appears that rather than expression of neural genes that would be species-specific, neural speciation is dictated and/or reflected by: (i) the whole number of neurons, (ii) the number and histological organization of specific neuronal populations and, most importantly, (iii) the establishment of species-specific inter-neuronal networks (also called synaptic networks or circuits), and (iv) the species-specific ability to mount or refine networks, notably during development, a process named synaptic plasticity. On this basis, I propose the term “neural repertoire” to designate the whole of neuronal populations and interneuronal synapses that characterize a given individual from a given species at a given time. From a molecular point of view, a neural repertoire is mostly reflected and dictated by the neuronal expression of specific sets of genes that are determined by speciation, developing stage, and the whole neuronal activity of the individual bearing such a neural repertoire at a given time. Since non-neuronal cells such as oligodendrocytes (the myelin-forming cells) and astrocytes are intimately associated to the functions of neurons including the completion of cognitive tasks, the name “neural repertoire” appears more appropriate than “neuronal repertoire.” Accordingly, genes expressed by oligodendrocytes and astrocytes, which are neural cells (i.e., in the sense of belonging to the nervous system), are also considered as shaping the neural repertoire of an individual.

## Brain superautoantigens: connections between neural and T-cell repertoires

### Physiological connections between neural and T-cell repertoires

The brain superautoantigens theory proposes that, in any species endowed with an adaptive immune system, the neural repertoire of an individual is constantly perceived by the immune system via a two-step process: (i) the capture of neural antigens by antigen-presenting cells, (ii) the activation of cognition-promoting T-cells that recognize such presented autoantigens. In this scheme, not all neural proteins may elicit an adaptive autoimmune response and only a subset of brain-derived antigens may be considered as “brain superautoantigens.” Of note, the term superautoantigens should not be confused with the one of superantigens which, in immunology textbooks, designates a particular group of microbial antigens endowed with a unique ability to stimulate a large population of T-cells via molecular mechanisms that do not involve antigen presentation (Marrack and Kappler, 1990). This being kept in mind, there are 3 main features that may distinguish a brain superautoantigen from any brain-derived protein harboring potential antigenicity:

- a brain superautoantigen derives from a neural protein that is abundant and exhibits a high renewal rate
- a brain superautoantigen derives from a neural protein that is crucially involved in main neuronal functions such as synaptic transmission, synaptic plasticity, neurite extension or axonal tranport
- a brain superautoantigen presented under *ad hoc* conditions elicits the T-cells synthesis of neuroprotective molecules

Overall, one may consider that the neural repertoire somehow “express” brain superautoantigens that are recognized by the T-cell repertoire. Importantly, the extent to which brain superautoantigens may elicit a neuroprotective T-cell response does not rely only on the intrinsic features of such autoantigens. In particular, it is proposed that the evolutionary-determined speciation of the immune system scales the level of cognition-promoting autoimmunity in a given species. In other words, two species harboring similar neural repertoires may differ with regard to the level of cognition-promoting autoimmunity afforded by their respective immune systems. As explained thereafter, such differences may notably rely on the fine evolution of genes playing a major role in antigen presentation and/or the control of autoimmunity. Also, when considering two individuals from the same species, their respective levels of cognition-promoting autoimmunity may depend on their respective neural repertoires (shaped by their neuronal activity, synaptic plasticity, levels of synaptic transmission between specific neuronal populations…) and their respective immune status (immunodepression, immunodeficiency, systemic immune activation…).

### Pathological connections between neural and T-cell repertoires

**Figure 2:**
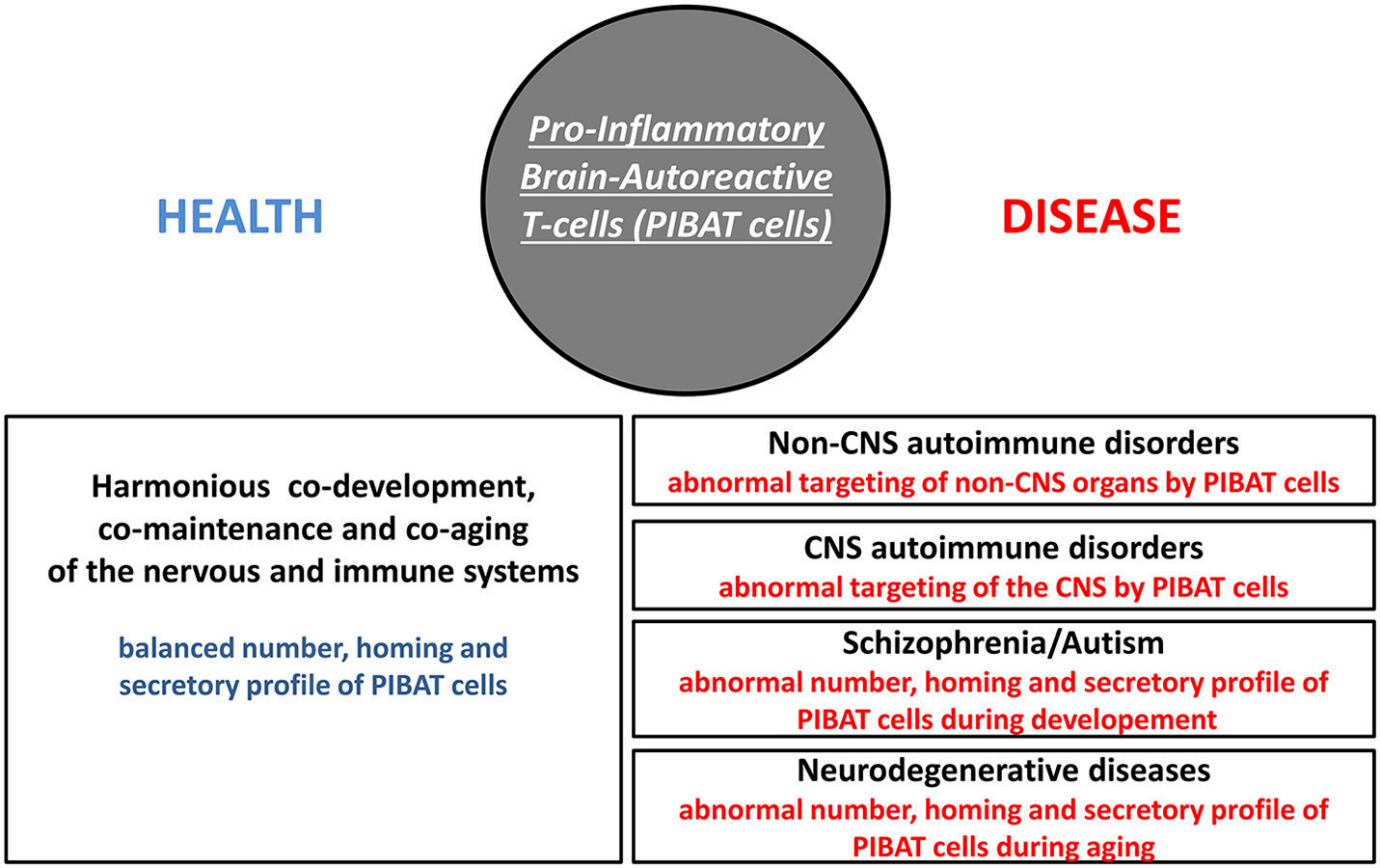
Pro-inflammatory brain-autoreactive T-cells (PIBAT cells) in health and disease. Under physiological conditions, the number, secretory profile and homing properties of pro-inflammatory brain-autoreactive T-cells (PIBAT cells) supports finely-tuned cognition-promoting autoimmunity. In particular, PIBAT cells exert at distance trophic effects on neurons via cytokines and neurotrophins that are appropriately synthesized (qualitatively and quantitatively). Cognition-promoting autoimmunity allows an overall harmonious co-development, co-maintenance and co-aging of the nervous and immune systems. It is proposed that alterations in the number, secretory profile and/or homing behavior of PIBAT cells are involved in the pathophysiology of CNS autoimmune disorders, non-CNS autoimmune disorders, psychiatric diseases and neurodegenerative conditions.

In *Homo sapiens*, cognition-promoting autoimmunity is an evolutionary-determined advantage that brings with it a higher susceptibility to several categories of disorders including organ-specific autoimmune diseases, schizophrenia, autism and neurodegenerative conditions. Since neuroprotective brain-autoreactive T-cells were shown to harbor functional features of pathogenic T-cells, fine regulatory mechanisms are needed to control the number, homing properties and cytokine profile of such cells. This crucial regulatory function is currently considered to be essentially mediated by a subset of T-cells called regulatory T-cells (Treg cells or Tregs). Indeed Tregs dampen the activation of potentially pathogenic T-cells via a mechanism that is, at least in part, antigen-specific (Levine et al., 2017). Such a process notably relies on a presentation of the targeted antigen to pro-inflammatory T-cells and Tregs concomitantly (Liu et al., 2015). As discussed more thoroughly in a later section, brain-autoreactive Tregs are likely to play an essential role in maintaining a physiological equilibrium between cognition-promoting autoimmunity and pathological autoimmunity. Irrespective of the putative functions of brain-autoreactive Tregs, one may envision 3 main categories of pathological processes that might be induced by qualitative or quantitative alterations of pro-inflammatory brain-autoreactive T-cells (thereafter referred to as PIBAT cells) i.e., cells which, under physiological conditions, exert otherwise cognition-promoting functions (Figure 2).

#### Pathologies potentially induced by an expansion and/or an altered homing of pro-inflammatory brain-autoreactive T-cells (PIBAT cells).

Since an important part of cognition-promoting autoreactive T-cells express features of pro-inflammatory pathogenic T-cells it appears probable that any substantial CNS invasion by such cells would provoke a CNS autoimmune disorder. We proposed that paraneoplastic syndromes (PNS) are secondary to an infiltration of the CNS parenchyma by PIBAT cells that pre-exist to the PNS-triggering cancer (Nataf, 2017a,b). In this scheme, PIBAT cells directed against a specific set of synaptic autoantigens would be subjected to quantitative and/or qualitative alterations accompanying the anti-tumoral adaptive immune response. More specifically, the expression of synaptic antigens by tumoral cells would trigger the expansion of pre-existing PIBAT cells. That PNS very rarely target non-CNS organs would be explained by the lack or scarcity of pre-existing pro-inflammatory T-cells directed against non-neural tumoral antigens. However, as a matter of fact, despite the Sword of Damocles that PIBAT cells place over the CNS, the incidence of paraneoplastic syndromes remains extremely low. Similarly, the incidence of multiple sclerosis, the main CNS autoimmune disorder, is itself relatively low. These observations are not incompatible with the brain superautoantigens theory for essentially 2 reasons: (i) as explained above, PIBAT cells may exert at distance cognition-promoting effects notably when activated in the lymph nodes and (ii) the blood-brain barrier drastically restricts the migration of immune cells from blood to brain (Engelhardt and Ransohoff, 2012; Obermeier et al., 2013). Accordingly, the major risk run by the existence of cognition-promoting PIBAT cells is not CNS autoimmunity but indeed non-CNS autoimmunity. This apparently paradoxical view is supported by a reappraisal of the recent literature in neurosciences and immunology showing that a large share of the autoantigens targeted in non-CNS autoimmune disorders are in fact brain autoantigens. Such autoantigens harbor the above mentioned features of brain superautoantigens and are enriched in CNS and non-CNS locations (Nataf, 2017a,b). In this pathophysiological scheme, the expansion and/or altered homing of PIBAT cells would be responsible for the pathological targeting of brain superautoantigens expressed in extra-CNS locations. Table 2 provides a summary of the main experimental and epidemiological arguments supporting the brain superautoantigens theory in the context of pathological autoimmunity. Table 3 is a proposed non-exhaustive list of brain superautoantigens involved in organ-specific autoimmune diseases.

**Table 2:** Five data-driven arguments support the brain superautoantigens theory.

**1) Myelin- or synapse-derived autoantigens harbor immunogenic features**
^*^ abundance ^*^ high renewal rate ^*^ expression in a context of physiological inflammation
**2) Major antigens in non-CNS autoimmune diseases belong to the synaptic compartment**
^*^ GAD65 ^*^ AchR ^*^ HSPA5 ^*^ HSP60 ^*^ SnRNP ^*^ gangliosides ^*^ TH ^*^ AchE ^*^ collagen IV ^*^ laminin
**3) Cognitive alterations are frequently observed in non-CNS autoimmune diseases**
**4) CNS autoimmunity essentially targets myelin- or synapse-derived autoantigens**
**5) “Incidental” autoimmunity essentially targets myelin- or synapse-derived autoantigens**
^*^ paraneoplastic syndromes ^*^ Guillain-Barre syndrome ^*^ ASIA ^*^ Post-vaccination narcolepsy

*GAD65, Glutamate decarboxylase 65 (GAD2), a synaptic enzyme that catalyzes γ-aminobutyric acid (GABA) synthesis from glutamate, is also the main targeted autoantigen in type I diabetes; AchR, acetycholine receptor is a major autoantigen in myasthenia gravis; HSPA5, heat shock protein family A (Hsp70) member 5 is targeted in rheumatoid arthritis; HSP60 (HSPD1), heat shock protein family D (Hsp60) member 1 is also an autoantigen in rheumatoid arthritis; SnRNPS, Small nuclear ribonucleoproteins are the main autoantigens in systemic lupus erythematous (SLE) and in Sjögren syndrome; gangliosides are autoantigens in Guillain-Barre syndrome; TH, tyrosine hydroxylase is one of the targeted autoantigen in vitiligo; AchE, acetylcholinesterase harbor high similarities with the acetylcholinesterase domain of thyroglobulin, a main autoantigen in Hashimoto's disease (autoimmune thyroiditis). ASIA, Autoimmune/inflammatory syndrome induced by adjuvants. Post-vaccination narcolepsy is a secondary effect that was observed following the 2009 H1N1 vaccination campaign. This Table is reproduced with permission and slight modifications from the chapter “Brain superautoantigens: connections between immune and neural repertoires” published in the e-book “Brainimmune” (Nataf, 2017a)*.

**Table 3:** Major autoantigens targeted in non-CNS organ-specifc autoimmune disorders belong to the synaptic compartment.

**Autoantigen**	**Autoimmune disease**	**Synaptic localization**	**Neuronal functions**
GAD65	Diabetes type I	+	GABAergic neurotransmission
AchR	Myasthenia gravis	+	Cholinergic neurotransmission
HSPA5^a^	Rheumatoid arthritis	+	Glutamatergic neurotransmission
HSP60^b^	Rheumatoid arthritis	+	Motor functions
ribosomes^c^	SLE	+	Synaptic plasticity neurotransmission
snRNPs^d^	SLE, Sjögren's syndrome	?	Cognition, motor functions?
hnRNPs^e^	SLE, Sjögren's syndrome rheumatoid arthritis	+	Synaptic plasticity
collagen IV	Glomerulonephritis	+	Synaptic plasticity
laminin	Glomerulonephritis autoimmune dermatoses	+	Synaptic plasticity
TH	Vitiligo	+	Dopaminergic neurotransmission
MCHR1	Vitiligo	+	Energy balance, sleep, mood
AchE^e^	Autoimmune thyroiditis (Hashimoto's disease)	+	Cholinergic neurotransmission
gangliosides^f^	Guillain-Barre syndrome	+	Glutamatergic neurotransmission

*GAD65, glutamate decarboxylase 65 (GAD2), a synaptic enzyme that catalyzes γ-aminobutyric acid (GABA) synthesis from glutamate; AchR, acetycholine receptor; HSPA5, heat shock protein family A (Hsp70) member 5; HSP60 (HSPD1), heat shock protein family D (Hsp60) member 1; snRNPS, small nuclear ribonucleoproteins; hnRNPS, heterogenous nuclear ribonucleoproteins; TH, tyrosine hydroxylase; MCHR1, melanin-concentrating hormone receptor 1; AchE, acetylcholinesterase; SLE, systemic lupus erythematous*.

a *HSPA5 is a major component of the synaptic glutamate receptor complex*.

b *HSP60 is a mitochondrial chaperone molecule; a high density of mitochondria is observed in synaptic boutons and mitochondria play an important role in the control of synaptic neurotransmitter release; accordingly, mutations in HSP60 (HSPD1) are responsible for autosomal recessive spastic paraplegia 13*.

c *Ribosomes are enriched in synaptic boutons and in the postsynaptic compartment, allowing local synthesis of proteins involved in neurotransmission and synaptic plasticity*.

d *In neurons, snRNPs (small nuclear ribonucleoproteins) are protein interactants of RNA-binding proteins playing a crucial role in cognition and/or motor functions; these include FRMP (fragile × mental retardation protein), SMN (survival of motor neurons) and ELAVL1(ELAV like RNA binding protein 1). A variant of the snRN gene TROVE2 (the Ro60-coding gene) is associated with specific memory skills*.

e *hnRNPS are abundant in the pre-synaptic and post-synaptic compartments of neurons and have been involved in synaptic plasticity*.

f *Thyroglobulin, a main autoantigen in Hashimoto's disease, bears an acetylcholinesterase domain that is essential to both immunogenicity and functions of thyroglobulin. f: gangliosides are highly abundant on the outer layer of neuronal membranes and are involved in multiple neuronal functions including trafficking of glutamate receptors in the postsynaptic membrane; human mutations in ganglioside biosynthetic enzymes are responsible for autosomic disorders that translate into intellectual disability and, less frequently, epilepsy. This table is reproduced with permission from the chapter “Brain superautoantigens: connections between immune and neural repertoires” published in the e-book “Brainimmune” (Nataf, 2017a)*.

#### Pathologies potentially induced by a decreased number, a decreased activation state and/or an altered homing of pro-inflammatory brain-autoreactive T-cells (PIBAT cells)

Considering that cognition is supported by autoimmune mechanisms implies that pathologies during which cognition is abnormal may be possibly linked to a decreased number, a decreased activation state and/or an altered homing of cognition-promoting T-cells i.e., PIBAT cells. As exposed elsewhere (Nataf, 2017b), this may be notably the case for psychiatric disorders such as schizophrenia and autism, two conditions characterized by the co-occurrence of cognitive and immune alterations Also, part of the link between aging and an increased susceptibility to neurodegenerative diseases may rest on a process of immunosenescence which may translate into a decreased number, a decreased activation state and/or an altered homing of brain-autoreactive and cognition-promoting T-cells (Ron-Harel and Schwartz, 2009). However, as described below, immunosenescence may not be the only immune-related mechanism involved in neurodegeneration.

#### Pathologies potentially induced by the alteration of brain superautoantigens immunogenicity

The cytokine profile and homing properties of any antigen-specific activated T-cells are, at least in part, determined by the biochemical intrinsic features harbored by the processed antigen (Roche and Furuta, 2015). Biochemical alterations of a brain superautoantigen may thus result in functional alterations of the PIBAT cells targeting such an antigen. This may hold relevance in the context of neurodegenerative disorders. Indeed, such conditions are driven by the pathogenic aggregation and/or misfolding of major neuronal proteins that harbor features of brain superautoantigens. This is notably the case for amyloid beta and tau proteins, both involved in Alzheimer's disease (Ross and Poirier, 2004; Spillantini and Goedert, 2013), and for alpha-synuclein involved in Parkinson's disease (Lee et al., 2014). How aggregated or misfolded forms of such antigens may alter the functions of pre-existing PIBAT cells requires further attention.

#### Neuroimmune co-pathologies

**Figure 3:**
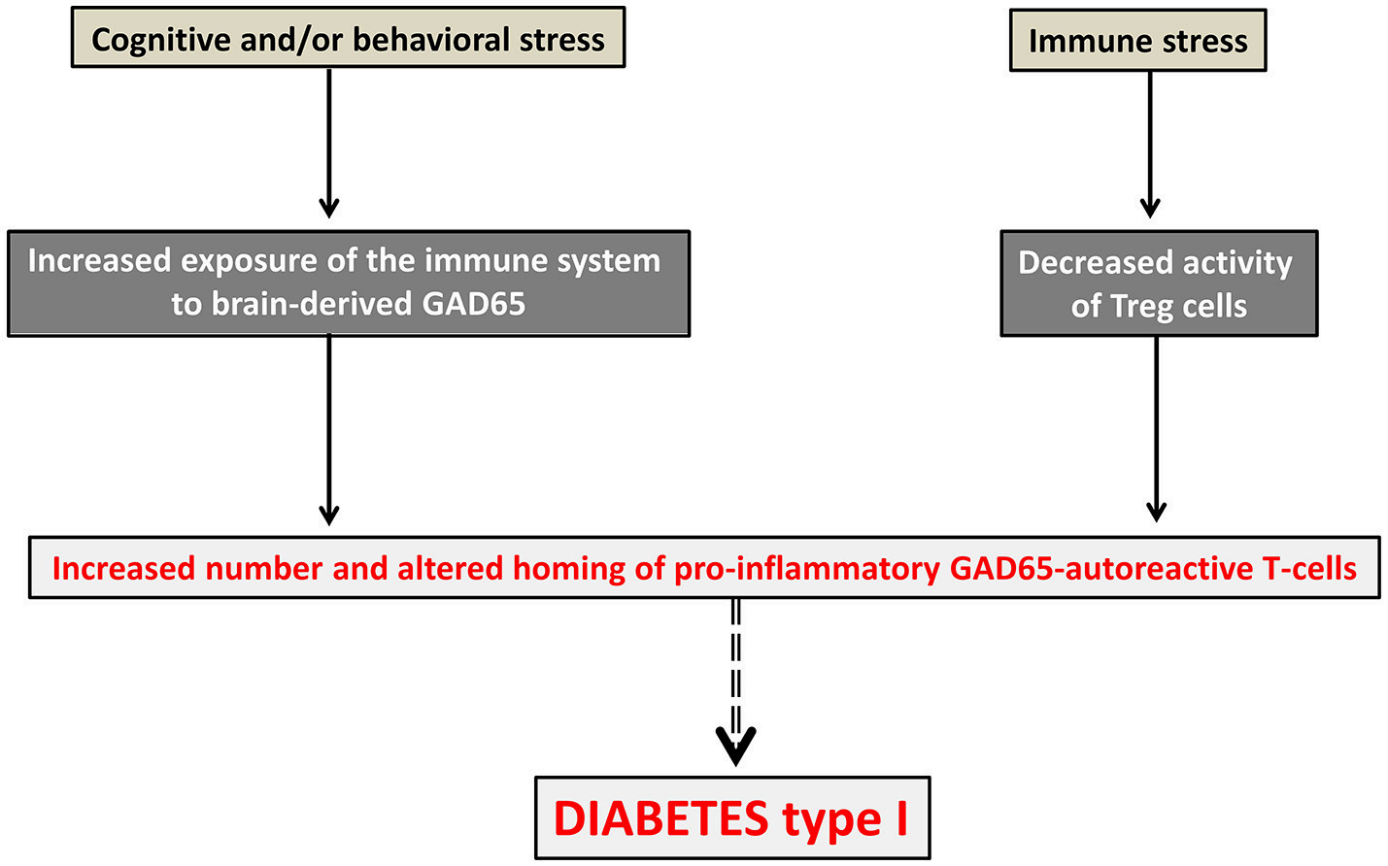
Diabetes type 1 viewed as a neural disorder. In this pathophysiological scheme, pathological autoimmunity against GAD65, the main targeted autoantigen in diabetes type 1, would result from both immune and neural alterations occurring concurrently. An immune stress such as, for instance, a viral infection would lead to a drop of Tregs. In parallel, a cognitive or behavioral stress (for instance cognitive overstimulation or mood imbalance) would be responsible for an increased exposure of the immune system to the brain superautoantigen GAD65. Pathological autoimmunity against pancreatic islet cells expressing GAD65 antigen would then result from an increased number, increased activation state and altered homing of GAD65 autoreactive T-cells.

Assuming the existence of tight connections between neural and T-cell repertoires should lead to re-consider the pathophysiology of disorders that are usually viewed as primarily neurological or primarily immunological. The term “neuroimmune co-pathologies” was proposed to describe the interdependence between neural and T-cell repertoires in a large range of pathological conditions (Nataf, 2017a). These include autoimmune disorders, paraneoplastic syndromes, neurodegenerative conditions, schizophrenia, autism and possibly other psychiatric diseases. Whether such interdependence also applies to neural and antibody repertoires is an important issue that would require specific assessment. In any case, important pathophysiological and therapeutic implications may ensue from the demonstration of interconnections between neural and T-cell repertoires. With regard to pathophysiology, such tight interconnections would imply that the *primum movens* of autoimmune disorders might be, at least in part, of neural origin. This means that alterations of the neural repertoire (leading for instance to the overexpression of a particular set of brain superautoantigens) might be responsible for alterations of the T-cell repertoire directed against those brain superautoantigens. In turn, such alterations (for instance an expansion and/or an altered homing of T-cells directed against those brain superautoantigens) would lead to the emergence of pathological autoimmunity [as described in section Pathologies Potentially Induced by an Expansion and/or an Altered Homing of Pro-inflammatory Brain-Autoreactive T-cells (PIBAT Cells)]. An example is given in Figure 3 with GAD65 (glutamate acid decarboxylase) the main autoantigen in diabetes type I and also an essential synaptic enzyme allowing the generation of the neurotransmitter GABA (gamma-Aminobutyric acid). Conversely, a primary process altering the T-cell repertoire directed against brains superautoantigens [as described in sections Pathological Connections between Neural and T-cell Repertoires and Pathologies Potentially Induced by an Expansion and/or an Altered Homing of Pro-inflammatory Brain-Autoreactive T-cells (PIBAT Cells)] could constitute the *primum movens* of neuropsychiatric or neurodegenerative disorders.

#### Therapeutic implications

Finally, with regard to therapy, it could be considered that vaccination strategies allowing to manipulate the T-cell repertoire against brain superautoantigens might support a cognition-promoting and neuroprotective effect. Indeed, animal studies previously showed that vaccination against brain antigens, in particular MBP, could afford clinical benefits under distinct neurological conditions. In another hand, manipulating the neural repertoire could be envisioned for the treatment of autoimmune diseases. Mental or sensorimotor tasks could be performed in order to modulate (increase or decrease) the levels at which specific brain superautoantigens are expressed in the CNS and thus captured by antigen-presenting cells. The ensuing modulation of the T-cell repertoire would then allow to dampen autoimmunity against brain superautoantigens that localize in non-CNS histological sites [as described in section Pathologies Potentially Induced by an Expansion and/or an Altered Homing of Pro-inflammatory Brain-Autoreactive T-cells (PIBAT Cells)].

### Brain superautoantigens and the co-development/co-evolution model

**Figure 4:**
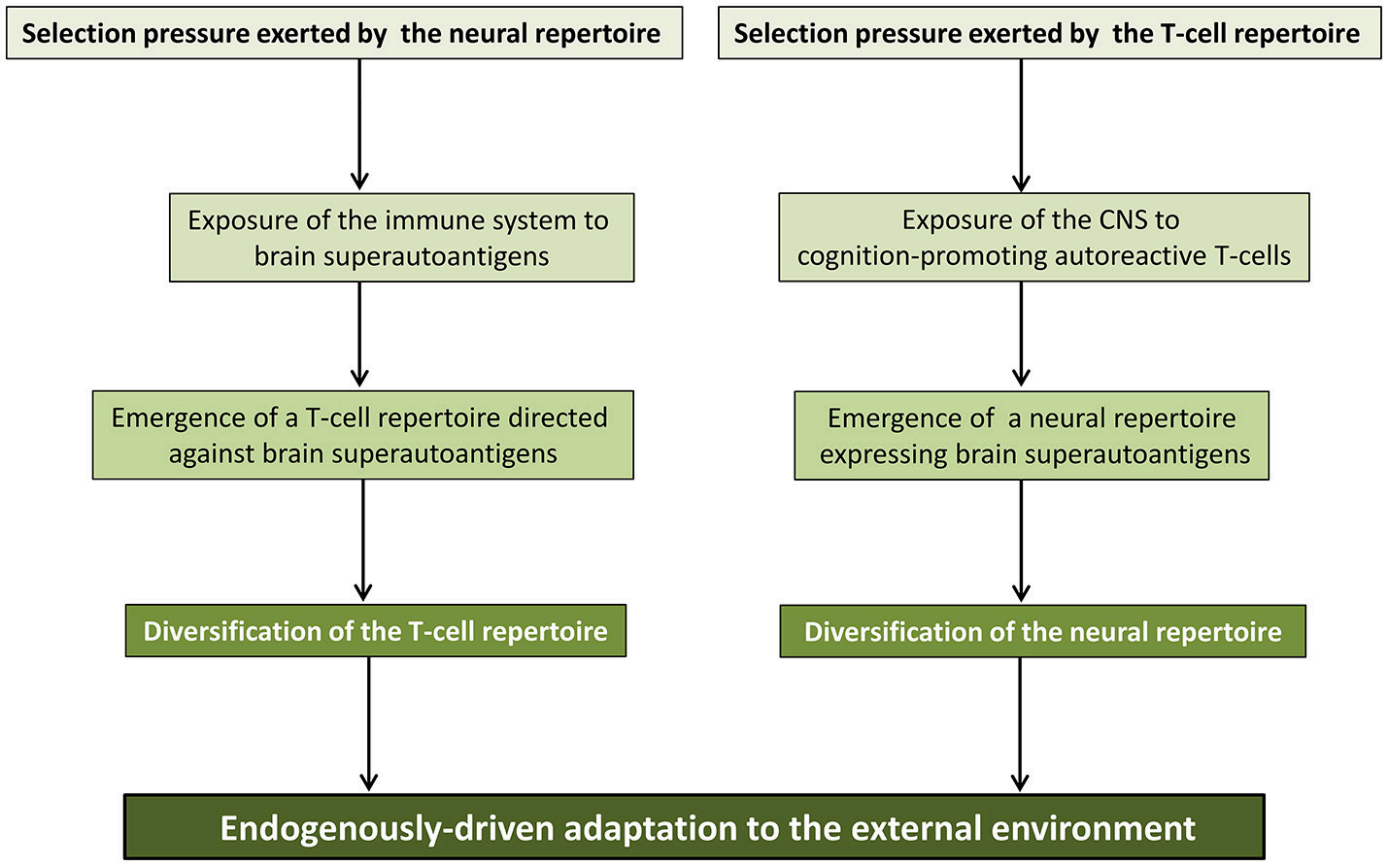
Co-evolution/co-development of the neural and immune repertoires. The co-development/co-evolution model proposes that during evolution, neural and T-cell repertoires have exerted mutual selection pressures. In one hand, the T-cell repertoire directed against brain superautoantigens may have favored the emergence of a neural repertoire expressing brain superautoantigens. On the other hand, the neural repertoire expressing brain superautoantigens may have favored the emergence of a T-cell repertoire directed against brain superautoantigens. As a result, evolution of the immune and nervous systems, the two main systems allowing the sensing of and adaptation to the external environment would have been, at least in part, endogenously-driven. This figure is reproduced with permission and slight modifications from the chapter “Brain superautoantigens: connections between immune and neural repertoires” published in the e-book “Brainimmune” (Nataf, 2017a).

In the brain superautoantigens theoretical frame, the term “co-development/co-evolution” describes the developmentally-regulated and evolutionary-determined selection pressures that T-cell and neural repertoires may mutually exert via brain superautoantigens (Nataf, 2017b). As shown in Figure 4, in this two-way process, the evolutionary-determined emergence of neurons expressing specific immunogenic antigens (brain superautoantigens) has exerted a selection pressure on immune genes shaping the T-cell repertoire. Such a selection pressure on immune genes has translated into the emergence of a finely tuned autoimmune T-cell repertoire that promotes cognition. In another hand, the evolutionary-determined emergence of brain-autoreactive T-cells has exerted a selection pressure on neural genes coding for brain superautoantigens. Such a selection pressure has translated into the emergence of a neural repertoire expressing brain superautoantigens. Thus, the endogenously-driven mutual selection pressures of T-cell and neural repertoires would have participated in shaping speciation via mechanisms that are not primarily determined by the external environment. In other terms, divergence between species that have been submitted to a similar environment during a similar period of time would be explained, at least in part, by a divergence of their internal neuroimmune environment and the subsequent divergence of their neuroimmune skills for the sensing of and adaption to the external environment. Of note, it should be kept in mind that multiple evolutionary-determined mechanisms may have supported the emergence of cognition-promoting brain-autoreactive T-cells. These notably include: (i) mechanisms allowing an increased exposure of the immune system to brain superautoantigens and (ii) the acquisition of MHC class II genes that permit brain superautoantigesn to be efficiently presented to T-cells. Finally, it is also suggested that in any species endowed with an adaptive immune system, neural and T-cell repertoires exert mutual supports and selection pressures during development. Such a process would allow the co-establishment and co-maintenance of the neural and T-cell repertoires. This last point is of particular interest in the context of developmental brain disorders such as autism and schizophrenia.

## Toward an evolutionary genetics of cognition-promoting autoimmunity

The notion of co-evolution between the nervous and immune systems and, more specifically, between neural and T-cell repertoires suggests that genes involved in adaptive immune responses may have been crucial for cognitive evolution. In this regard, the Foxp and HLA-DR families of genes may have played a major role.

### The foxp family of genes: roles in cognition and autoimmunity

**Figure 5:**
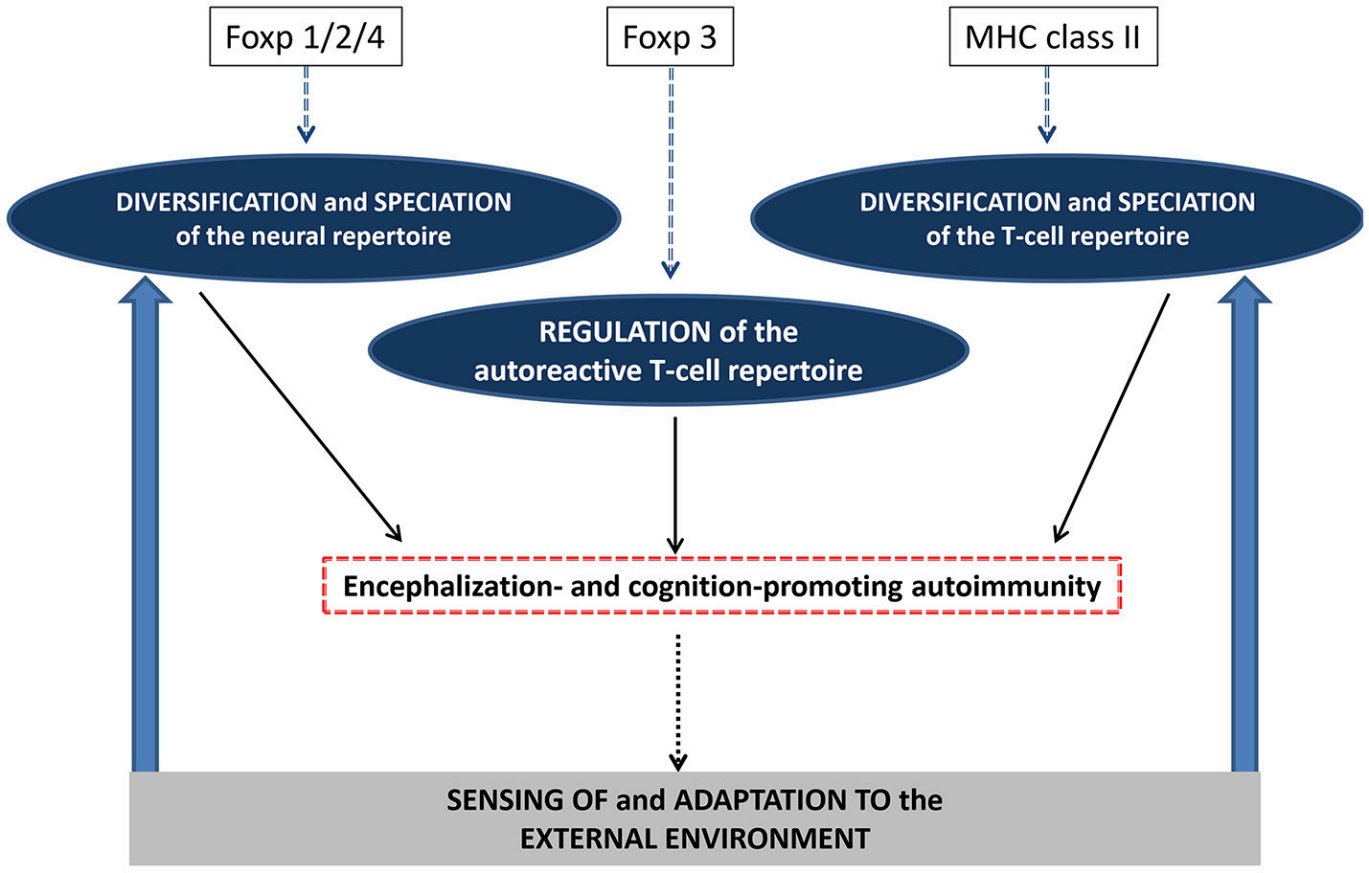
Autoimmunity and the evolutionary genetics of encephalization and cognition. It is suggested that evolution of the Foxp1/2/4 genes have not only contributed to a diversification of neural repertoire but have instructed what could be called a “speciation” of such repertoire i.e., the acquisition of species-specific features. In parallel, evolution of the MHC class II genes have contributed to the diversification and “speciation” of the T-cell repertoire, notably the autoreactive T-cell repertoire directed against brain superautoantigens. Finally, evolution of the Foxp3 gene may have allowed a species-specific control of cognition-promoting autoimmunity. These combined mechanisms would support an endogenously-driven and species-specific processes which would shape encephalization and cognition. By this mean, external cues exerting a selection pressure on a given species would be filtered by the internally-fueled sensing skills of this species. In turn, perceived externals cues would further instruct the diversification and speciation of the neural and T-cell repertoires.

In mammals, the Foxp family of transcription factors derive from a common ancestor gene and comprises 4 members named Foxp1 to Foxp4. Interestingly, Foxp1, Foxp2, and Foxp4 are all involved in central nervous system (CNS) development and functions. In particular, disruptions of Foxp2 or Foxp1 give rise to rare human CNS disorders that clinically share an impairment of expressive language (Bacon and Rappold, 2012). Indeed, Foxp2 is the only gene whose alteration provoke a pure speech disorder with Mendelian inheritance (Lai et al., 2001, 2003). Foxp4 is also highly expressed in the brain and form functional heterodimers with Foxp2 or Foxp1 (Sin et al., 2015). Foxp3, unlike Foxp1/2/4, is not involved in brain development and cognition but is the main transcription factor driving the development of Treg cells (Ramsdell and Ziegler, 2014). As described earlier, an essential function of Tregs is to suppress pro-inflammatory T-cell responses directed against “self” antigens, and to drive the resolution of immune responses that would fuel excessive or unneeded inflammation. Accordingly, genetically-determined alterations of Foxp3 are responsible for a severe disorder called “Immunodysregulation, polyendocrinopathy, enteropathy, X-linked” (IPEX) which clinically translates into autoimmune conditions affecting the gut (autoimmune enteritis), skin (atopic eczema), pancreas (diabetes type I) and thyroid (Michels and Gottlieb, 2010). That a lack of Tregs induce pathological autoimmunity further confirms that autoimmunity is not absent under physiological conditions but is indeed regulated. As previously proposed (Schwartz and Kipnis, 2002), a finely tuned activity of Tregs allows the risks of pathological autoimmunity to not overcome the needs for physiological autoimmunity. While the apparent functional divergence between Foxp1/2/4 and Foxp3 may result from an aphazard gene evolution, the brain superautoantigens theory offers a comprehensive evolutionary scheme in which: (i) Foxp1/2/4 would have favored the development and maintenance of cognition-related neural repertoires expressing brain superautoantigens while (ii) Foxp3 would have allowed the control and fine-tuning of cognition-promoting immune repertoires directed against brain superautoantigens (Figure 5). In other terms, cognitive evolution in the homo genus would have resulted from an ideal compromise between the Foxp1/2/4-driven emergence of neural repertoires supporting human-specific cognitive functions and the Foxp3-regulated activity of cognition-promoting immune repertoires. Favoring this view, subtle inter-species differences in the coding sequences Foxp2 and Foxp3 were shown to profoundly impact the neural and immune transcriptional functions of Foxp2 and Foxp3 respectively (Enard et al., 2002; Zhang et al., 2002; Konopka et al., 2009; Andersen et al., 2012; Ayub et al., 2013). This observation suggests that Foxp1/2/4 may have contributed not only to a diversification of neural repertoires across evolution but somehow to a “speciation” of such repertoires (Figure 5). In parallel, the regulation of cognition-promoting autoimmunity exerted by Foxp3 might have been scaled by species-specific features of the Foxp3 gene. Finally, it is worth noting that, unexpectedly, Foxp3 and Foxp1/2/4 were also assigned neural and immune functions respectively. Thus, (i) Foxp3 is expressed in glial cells and inhibit inflammation-induced neuronal excitability (Wang et al., 2017), (ii) Foxp1 is indispensable to the proper development of B-cells (van Keimpema et al., 2014, 2015), (iii) Foxp2 expression is a prognosis factor in large B-cell lymphomas (Wong et al., 2016) and (iv) Foxp4 is involved in T-cell recall responses against microbial antigens (Wiehagen et al., 2012). Overall, the whole Foxp family of genes has possibly exerted a key evolutionary leverage force on cognitive evolution.

### The HLA-DR family of genes: roles in autoimmunity and cognition

Antigen-specific activation of CD4 T-cells requires that antigen-derived peptides are presented at the outer cell surface of APCs in a molecular pocket formed by MHC-class II molecules. An MHC molecule is formed by a heterodimer composed of an alpha and a beta chain. In humans, there are 3 main categories of MHC class II molecules, namely HLA-DR, HLA-DQ, and HLA-DP, that are all encoded by genes located on the short arm of chromosome 6. Interestingly, in primates, genes coding for MHC-class II molecules have been submitted to a high selective pressure (Erlich and Gyllensten, 1991; Schwaiger and Epplen, 1995; Andersson, 1998). Notably, the extensive allelic diversity observed in the group of genes coding for HLA-DR beta chain (HLA-DRB genes) was shown to coincide with hominoid speciation (Erlich and Gyllensten, 1991; Schwaiger and Epplen, 1995; Andersson, 1998). Moreover, several genes coding for HLA-DR molecules result from a duplication process that occurred after the separation of humans with chimpanzee (Erlich and Gyllensten, 1991; Schwaiger and Epplen, 1995; Andersson, 1998). There is thus a high evolutionary-driven genetic plasticity and inter-individual diversity in the HLA-DR region. One may conclude that such a diversity results from an adaptation process to the microbial environment leading to the emergence of HLA-DR genes that confer to the host the most efficient abilities to present microbial-antigens to CD4 T-cells. Diversity could also be considered here as a mean to combat efficiently, at the scale of the whole human species, new challenges posed by emerging pathogens. Increased diversity in the HLA-DR genes is indeed likely to increase the odds that a group of individuals will bear “*ad hoc*” MHC molecules to combat new pathogens. However, one may argue that such a process would have had similarly occurred in other species. Again, an alternative explanation resides in considering that physiological autoimmunity and notably physiological autoimmunity directed against brain-derived antigens is a major evolutionary advantage that HLA-DR genetic diversification conferred to the human species. One argument supporting this assumption is given by results from whole genome-wide association studies (GWAS). When performing a survey of the EMBL data base of GWAS (the “GWAS catalog) (Welter et al., 2014), one may observe that a vast majority of the pathological or physiological traits that associate with specific polymorphisms of the HLA-DR genes relate with adaptive immunity and antigen presentation (Supplementary Material 1). As it can be expected, these notably comprise infectious diseases, malignant tumors and autoimmune pathologies. However, surprisingly, several brain disorders and behavioral/cognitive traits also associate with polymorphisms of the HLA-DR genes (Table 4). These include schizophrenia, Parkinson's disease, Alzheimer's disease and fronto-temporal dementia. In the same way, the genes coding for HLA-DO and HLA-DM molecules, which are critically involved in the selection of antigen-derived peptides that associate with MHC-class II molecules (Yi et al., 2010; Álvaro-Benito et al., 2015), harbor a genetic diversity that links antigen presentation to psychiatric disorders and to behavioral/cognitive traits (Table 4). Overall, in the Homo genus, the diversification of genes involved in antigen-presentation to CD4 T-cells might have thus supported the development of cognition-promoting adaptive immune responses directed against specific sets of brain superautoantigens. Moreover, in *Homo sapiens*, the impact of cognition-promoting autoimmunity on the development and maintenance of neural repertoires would have reached the highest level of sophistication among primates. Coming with this evolutionary advantage, the need for a properly-regulated cognition-promoting autoimmunity would have also exposed *Homo sapiens* to the risks of developing psychiatric or neurodegenerative disorders. In this scheme, cognition-related pathologies would be thus driven by inefficient or altered cognition-promoting physiological autoimmunity (Baruch et al., 2016; Schwartz and Deczkowska, 2016) and by pathological autoimmunity directed against brain superautoantigens (Brochard et al., 2008; Laurent et al., 2017). How pathological autoimmunity may arise from physiological autoimmunity is a point of great importance that goes beyond the scope of this article. Finally, beyond the Homo genus, the neuroimmune co-development/co-evolution model may apply to any species able to mount an antigen-specific immune response via the interaction between TCRs and antigen-derived peptides loaded on MHC class II (or MHC class II-like) molecules (Figure 5). Such species encompass a large share of vertebrates (Hirano et al., 2011, 2013).

**Table 4:** GWAS (genome-wide association studies) associate MHC class II genes with brain-related disorders or traits.

	**Brain disorders**	**Behavioral or cognitive traits**
HLA-DRA	Parkinson's disease (20711177; 22451204; 24511991)	
	Fronto-temporal dementia (24943344)	
HLA-DRB1	Alzheimer's disease (25188341; 24162737)	
	Schizophrenia (23212062; 19571809)	
	Parkinson's disease (27182965)	
HLA-DRB5	Fronto-temporal dementia (24943344)	
	Parkinson's disease (21292315; 27182965	
	Schizophrenia (26198764; 23974872)	
	Alzheimer's disease (25188341; 24162737	
HLA-DMA	Schizophrenia (26198764)	
	Psychiatric disorders^a^ (23453885)	
HLA-DMB	Schizophrenia (26198764)	
	Psychiatric disorders^a^ (23453885)	
HLA-DOA		Red wine liking (25758996)
		White wine liking (25758996)
		Neurocitism (27089181)
HLA-DOB	Schizophrenia (26198764)	
HLA-DQA1	Schizophrenia (23212062; 19571809)	
	Parkinson's disease (27182965)	
HLA-DQB1	Essential hypersomnia	
	Schizophrenia (26198764)	
	Parkinson's disease (25064009)	
HLA-DPA1		Neurocitism (27089181)
HLA-DPB1		Gait speed in old age (28077804)

*A survey of the EMBL data base of GWAS (the “GWAS catalog”) (Welter et al., 2014), was performed in order to list the known genetic associations between polymorphisms of MHC class II genes and brain-related disorders or traits. HLA pseudogenes identified as such in the NCBI (National Center for Biotechnology Information) “Gene” database were excluded from the analysis. Numbers in brackets indicate the PMID (PubMed Identifier) of the study from which associations were found*.

a *In this study, authors identified the risk loci with shared effects on five major psychiatric disorders: autism spectrum disorder, attention deficit-hyperactivity disorder, bipolar disorder, major depressive disorder and schizophrenia. No disease or trait association is reported in the GWAS catalog for the HLA genes: HLA-DRB3, HLA-DRB4, and HLA-DQB3. No brain-related disorder or trait association with is reported in the GWAS catalog for the HLA genes: HLA-DQB2, HLA-DPA2 and HLA-DPA3 (see Supplementary Material 1). The survey presented in this table was performed on the 07/18/2017*.

## Conclusions

### On the links between autoimmunity and cognitive evolution in the homo genus

The present paper proposes that autoimmunity directed against brain superautoantigens has been a major driving force of cognitive evolution in the Homo genus. To take the reasoning further in a somehow provocative opinion on cognitive evolution, several speculative ideas can be put forward (Table 5). First, it could be suggested that, in *Homo sapiens*, the high diversity of genes coding for MHC class II molecules (and possibly other immune-related molecules) is, at least in part, responsible for a high diversity of human cognitive skills and behaviors. Just as the advantages provided by MHC diversity with regard to immune defenses against pathogens, MHC diversity and, overall, the diversity of immunogenetic backgrounds would ensure that, at the scale of the whole species, cognitive challenges can be collectively overcome via cognitive cooperation and complementarity. Another speculation that may deserve interest is the bridge which might be drawn between the emergence of distinct primordial human languages and the existence of distinct primordial groups of *Homo sapiens* in which roughly similar MHC and immunogenetic backgrounds were shared. Could the diversity of human languages be primarily linked to the diversity of MHC and immune-related genes? Favoring this hypothesis, it is now admitted that besides migratory flows and the selection pressures exerted by distinct geographical environments, genomic diversity in modern humans was shaped by distinct levels of genetic flows (a process called introgression) between *Homo sapiens* and archaic hominins, notably *Homo neanderthalensis* (Green et al., 2010). Interestingly, a genetic legacy of Neanderthal introgression is the acquisition of regulatory variants controlling the expression of major immune-related genes (Quach and Quintana-Murci, 2017). These notably include IL-17A, the pro-inflammatory cytokine characterizing TH17 cells, and STAT2, which was recently shown to regulate the functional activity of the TH1 cytokine interferon-gamma (Ho et al., 2016).

**Table 5:** Prospective ideas on autoimmunity-driven cognitive evolution.

**1) Diversity and complementarity of cognitive skills in the human species is in part determined by the diversity of immunogenetic backgrounds**
**2) Differences in immunogenetic backgrounds between separate populations of human ancestors may have been responsible for the emergence of distinct primordial languages**
**3) Physiological autoimmunity toward brain superautoantigens is qualitatively and quantitatively scaled to the cognitive skills of a given vertebrate species**
**4) In a large share of complex organisms, neuroimmune auto-perception (i.e., immune recognition of “self” neural patterns) is a general mechanism of cognition-promoting immunity**

### On the links between autoimmunity and cognitive evolution in vertebrates

Since mechanisms of adaptive immunity were identified in all vertebrates, including the jawless fishes, one may anticipate that physiological autoimmunity toward brain superautoantigens is qualitatively and quantitatively scaled to the cognitive skills of a given vertebrate species. Irrespective of the vertebrate species considered, potential drawbacks of the brain superautoantigens theory comprise the current lack of experimental data demonstrating a cognition-promoting role of autoreactive B-cells and autoantibodies (Nataf, 2017b).

### On the links between autoimmunity and cognitive evolution in invertebrates

Another important criticism comes from the obvious notion that cognition, behavioral complexity and neuroimmune interconnection are far from being the preserve of species endowed with an adaptive immune system. Invertebrates such as for instance the medicinal leech, exhibit finely-tuned neuroimmune interactions (Tasiemski and Salzet, 2017) and proceed to decision-makings when executing complex locomotor behaviors (Esch et al., 2002; Harley et al., 2013). To answer this valid criticism, one may argue that the dichotomy established between innate and adaptive immunity is possibly specious and that “custom-fit immunity” (Rimer et al., 2014) i.e., basically, adaptive immunity, operate in all forms of life (Rimer et al., 2014). On this basis, one may envision that any molecular system allowing immune cells to “recognize” neural molecular patterns and to provide a recognition-dependent trophic support to such neural cells represents a form of cognition-promoting autoimmunity. I propose the term of neuroimmune auto-perception to depict the whole of such immune mechanisms. As previously put forward by Jean Dausset, the discoverer of the MHC system, adaptive immunity could be thus “regarded as a late evolution from a self-recognition system” (Tasiemski and Salzet, 2017). In this extended view of autoimmunity, the neuroimmune co-development/co-evolution model might be relevant in a large range of species.

## Author contributions

SN elaborated and wrote this hypothesis and theory paper.

### Conflict of interest statement

The author declares that the research was conducted in the absence of any commercial or financial relationships that could be construed as a potential conflict of interest.
